# Controversias en Cardiología. Parte 2. ¿Debo tratar un síndrome coronario crónico de alto riesgo invasivamente desde el inicio? No, en la mayoría de casos

**DOI:** 10.47487/apcyccv.v1i4.98

**Published:** 2020-12-31

**Authors:** José María Carrasco Rueda, René Ricardo Rodríguez Olivares, Luis Murillo Pérez, Juan Manuel Muñoz Moreno, Alayo Lizana, Carlos Alberto

**Affiliations:** 1 Servicio de Cardiología clínica, Instituto Nacional Cardiovascular - INCOR. Lima, Perú. Servicio de Cardiología clínica Instituto Nacional Cardiovascular - INCOR Lima Perú

**Keywords:** Enfermedad Coronaria, Angina de Pecho, Revascularización Miocárdica, Coronary Disease, Terapeutics, Myocardial Revascularization

## Abstract

La definición de síndrome coronario crónico de alto riesgo varía según la prueba no invasiva empleada para evidenciar isquemia, lo que se consigue a través de un aumento del trabajo miocárdico y la demanda de oxígeno, generados con el uso de fármacos o el ejercicio. El abordaje inicial nos sumerge en la discusión de contextualizar en qué casos priorizar la terapia médica óptima inicial, frente al manejo invasivo inicial, con el objetivo de lograr la revascularización miocárdica. Este aspecto central será desarrollado en esta revisión, tomando en cuenta diversos estudios realizados hasta la fecha, en los que el manejo invasivo inicial no ha demostrado ser superior a la terapia médica óptima inicial en resultados clínicos relevantes como muerte o eventos adversos cardiovasculares mayores.

## Abstract (en)

The definition of the high-risk chronic coronary syndrome varies depending on the noninvasive test used to trigger ischemia. The triggering occurs through increased myocardial work and oxygen demand, either through exercise or drugs. The initial approach to the chronic coronary syndrome leads us to discuss in which cases to prioritize an optimal initial medical therapy or to perform an initial invasive procedure of myocardial revascularization. In this article, we analyze both approaches based on previous studies carried out to date, where the initial invasive management has not been shown to be superior to initial optimal medical therapy in outcomes such as death or major adverse cardiovascular events.

El síndrome coronario crónico (SCC), cuya principal manifestación clínica es la angina de pecho, es la forma de presentación más prevalente dentro del conjunto de enfermedades cardiovasculares ^1^. La Organización Mundial de la Salud (OMS) ha estimado que en el 2030 el número total de muertes por cardiopatía isquémica aumentará de 7,4 (2012) a 9,2 millones, y el costo en salud, solo en Estados Unidos de Norteamérica, ascenderá a 6 trillones de dólares para el 2027, considerando, además, que el costo por revascularización miocárdica asciende a más de 30 billones de dólares anuales ^1^^-^^4^.

La mayoría de los pacientes con SCC son asintomáticos y, por ende, infradiagnosticados, con riesgo de presentar complicaciones cardiovasculares debido a la ausencia de un manejo adecuado ^5^. Los objetivos terapéuticos se dirigen, principalmente, a disminuir el riesgo de eventos cardiovasculares agudos y mejorar la calidad de vida mediante la reducción de la sintomatología; metas que consigue el tratamiento médico óptimo (TMO) sin diferencias significativas según la evidencia actual respecto a la estrategia invasiva inicial ^6^^-^^10^. Por ello, el objetivo de esta revisión es brindar información reciente sobre el beneficio del uso del TMO como estrategia inicial conservadora en pacientes con SCC de alto riesgo.

## Diagnóstico

Dentro de la evaluación del SCC, uno de los pasos iniciales es asignar la probabilidad pretest a todo paciente con sospecha diagnóstica. Este enfoque ha demostrado sobrestimación del riesgo debido al desarrollo de pruebas diagnósticas más precisas; por ello, algunos ensayos clínicos y la vigente guía europea de SCC han actualizado estos valores validando su uso con nuevos esquemas de evaluación (the Updated Diamond-Forrester score [UDF], Coronary Artery Disease Consortium Clinical Score [CAD2]) ^11^^-^^13^. No obstante, el National Institute for Health and Care Excellence (NICE) recomienda abandonar la probabilidad bayesiana y guiar la decisión de acuerdo con la presencia o no de angina, recomendación que aún posee escaso soporte científico ^14^^,^^15^.

La elaboración detallada de la historia clínica basada en la presencia y características del dolor anginoso, así como los síntomas equivalentes, son la clave de la evaluación diagnóstica inicial del SCC ^7^.

Las pruebas funcionales están diseñadas para detectar isquemia miocárdica a través de cambios en el electrocardiograma mediante prueba de esfuerzo físico, anomalías del movimiento de la pared ventricular por ecocardiografía de esfuerzo, o cambios de perfusión mediante tomografía computarizada por emisión de fotón único (SPECT). La isquemia es provocada bien por el aumento del trabajo miocárdico y la demanda de oxígeno, o por la heterogeneidad en la perfusión miocárdica por vasodilatación ^1^. La definición de alto riesgo es distinta según el estudio diagnóstico empleado: una mortalidad cardiovascular anual >3% se correlaciona con un escore de Duke ≤ -11 en la prueba de esfuerzo; hipocinesia o acinesia en ≥ 3 de 16 segmentos en la ecocardiografía de esfuerzo; y un área de isquemia del ventrículo izquierdo ≥10% en el SPECT ^6^^,^^16^.

La evaluación anatómica no invasiva mediante la angiografía tomográfica computarizada (ATC) evalúa adecuadamente la presencia y extensión de enfermedad arterial coronaria (ateroesclerosis) y provee información relevante sobre las características de esta (placas blandas de baja densidad, remodelado externo, placas mixtas, etc.) las cuales se asocian a mayor probabilidad de eventos adversos. Dentro de la evaluación por ATC, el uso del sistema de datos y reporte de la enfermedad coronaria (CAD-RAS) tiene un valor diagnóstico y pronóstico superior al *score* de calcio y añade información pronóstica sobre el riesgo de accidente cerebrovascular (ACV) ^7^.

La ATC comparada con las pruebas funcionales clásicas ha demostrado una mejor categorización del riesgo de acuerdo con la presencia y extensión de la aterosclerosis coronaria, por ello, el manejo de los pacientes con SCC guiados por este método se asocia con una mejoría clínica, y una reducción de muerte o infarto de miocardio (IM) no fatal ^17^^-^^21^.

Diversos estudios avalan el uso de la ATC como prueba diagnóstica, siendo un referente inicial el estudio CONFIRM ^22^ donde la presencia de estenosis moderada o severa fue menor a la esperada, según la estratificación realizada por probabilidad pretest. Años después, el estudio PROMISE ^23^^)^ demostró que la ATC es una alternativa relevante a las pruebas funcionales en riesgo bajo a intermedio de SCC; sin encontrarse diferencias significativas en mortalidad e infarto de miocardio. Un subanálisis del mismo estudio demostró que el *score* de calcio posee una capacidad discriminatoria de eventos primarios (mortalidad, IM no fatal, hospitalización por angina inestable) similar a las pruebas funcionales ^18^. El estudio SCOT - HEART^19^ evidenció que la ATC incrementa la certeza diagnóstica, mejora la decisión de intervención y reduce el riesgo de IM. Lo descrito está en concordancia a lo reportado en un metaanálisis y un registro danés, donde se observó un beneficio significativo de la ATC sobre otras pruebas funcionales, al presentar menor riesgo de IM, aumento en la indicación de revascularización miocárdica (RVM), y mayor asociación con el uso de estatinas, aspirina y procedimientos invasivos ^20^^,^^21^. Por ello, la ATC ha demostrado ser una prueba diagnóstica anatómica capaz de tener un impacto clínico en el manejo de pacientes con SCC, ello debido a su enfoque en la evaluación de la aterosclerosis y las características de la placa ^22^^,^^23^.

En los últimos años, tanto el estudio COURAGE ^24^ e ISCHEMIA ^25^ no encontraron asociación entre la determinación de isquemia en pruebas funcionales y la probabilidad de eventos cardiovasculares adversos mayores; por el contrario, la presencia de aterosclerosis severa multiarterial y, por ende, la carga aterosclerótica total, ha demostrado mayor asociación con el riesgo de eventos. Así lo describe un subestudio del COURAGE, en pacientes diagnosticados de SCC por imágenes de perfusión miocárdica al esfuerzo y ATC el cual demostró que la presencia de enfermedad coronaria severa por ATC era capaz de predecir la supervivencia a largo plazo, a diferencia de la isquemia severa documentada por perfusión miocárdica ^26^.

Aun así cabe resaltar que, las pruebas de estrés pueden contribuir a determinar el origen isquémico del dolor torácico y la severidad de la sintomatología, además podrían ser de utilidad en pacientes de riesgo bajo o cuando la calidad de la imagen tomográfica no es óptima por artefactos de movimiento, taquicardias refractarias y/o calcificación coronaria muy severa ^7^.

## Definición de terapia médica óptima en SCC

### Medidas no farmacológicas

Los cambios en los estilos de vida y la dieta balanceada son estrategias fundamentales en la prevención primaria y secundaria de eventos cardiovasculares, dentro de estos, se incluye realizar actividad física aeróbica de intensidad moderada al menos 30 min al día, ≥ 5 días/semana, control de peso, dieta (siendo la Pesco-mediterránea la más recomendable) y el abandono del tabaco que reduce hasta 36% el riesgo de mortalidad ^6^.

### Tratamiento farmacológico óptimo

El objetivo del tratamiento óptimo es el aumento de la supervivencia y mejoría de los síntomas, por ello la prevención de eventos cardiovasculares se centra en reducir la incidencia de eventos trombóticos agudos y en disminuir el desarrollo de disfunción ventricular a través de estrategias farmacológicas ^6^^,^^27^**(**Tabla 1**)**.

**Tabla 1:** Principales grupos de fármacos utilizados en el tratamiento médico conservador del síndrome coronario crónico

Medicamento	Mecanismo de Acción	Indicaciones
Betabloqueador	Efecto cronotropico e inotrópico negativo. Prolongacion de la diastole	Terapia de primera linea para alivio de dolor toracico. Historia de infarto de miocardio o FEVI menor a 40%
Nitratos	Vasodilatacion venosa y arterial	Manejo agudo y cronico de dolor
Bloqueadores de canales de calcio	Union a canales de calcio tipo L, genera vasodilatacion central y periferica	Terapia de primera linea para alivio de dolor toracico. Manejo cronico de dolor
Acido acetilsalicilico	Bloqueo irreversible de la ciclooxigenasa 1, previniendo la formacion de trombos	Prevencion secundaria de infarto de miocardio
Inhibidores P2Y12	Inhiben el receptor P2Y12, bloqueando la activacion plaquetaria	Prevencion secundaria de infarto de miocardio
Estatinas	Inhibidor competitivo de la HMG CoA	Dosis de alta intensidad (atorvastatina o rosuvastatina) disminuyen el LDL y mortalidad cardiovascular
IECAs	Inhibicion del sistema renina angiotensina aldosterona, generando una vasodilatacion arterial y venosa	hipertension arterial, diabetes mellitus o disfuncion de ventriculo izquierdo

FEVI= fracción de eyección de ventrículo izquierdo. LDL= lípidos de baja densidad. HMG CoA= Hidroximetilglutaril coenzima A. IECAS: Inhibidores de la enzima convertidora de angiotensina

El tratamiento antianginoso se centra en el control, alivio y disminución de síntomas relacionados con la angina, sea esta de presentación típica o atípica y, como consecuencia, mejora la calidad de vida del paciente. Fármacos como los betabloqueantes y los antagonistas de los canales de calcio dihidropiridínicos son la primera línea de tratamiento antianginoso al mejorar los síntomas; no obstante, su beneficio en la reducción de complicaciones cardiovasculares es discutible ^6^. Como manejo de segunda línea están los nitratos, ivabradina, ranolazina, nicorandil ^27^^-^^29^ o la trimetazidina; sin embargo, esta última utilizada a una dosis de 35 mg dos veces al día, posterior a una ICP exitosa, no logró influir en la recurrencia de angina ni en el desenlace primario (muerte, hospitalización por causa cardiaca) ^30^.

La antiagregación plaquetaria también es uno de los pilares en el tratamiento del SCC, ya que la activación plaquetaria y la trombosis son los desencadenantes de la mayoría de las complicaciones cardiovasculares en este grupo de pacientes. La antiagregación en monoterapia sigue siendo el tratamiento de elección en SCC, al prevenir la formación de trombos intracoronarios debido a la inhibición de la activación y agregación plaquetaria mediante el bloqueo irreversible de la ciclooxigenasa o la inhibición del receptor P2Y12 ^29^. Otra estrategia es el tratamiento combinado con anticoagulantes, como rivaroxabán (inhibidor del factor Xa) a dosis baja (2,5 mg dos veces al día), en pacientes en ritmo sinusal, demostrando beneficio en reducir la incidencia de complicaciones cardiovasculares mayores, con el atenuante de incrementar el sangrado no fatal ^6^^,^^31^.

Las estatinas, otro medicamento fundamental en el manejo médico del SCC, han demostrado una reducción significativa de LDL (cLDL <55 mg/dL) y reducción de eventos cardiovasculares no fatales en comparación con placebo. Cuando no se consigue el objetivo planteado en pacientes de muy alto riesgo cardiovascular (cLDL < 55mg/dL), se recomienda agregar un inhibidor de la absorción intestinal de colesterol (ezetimibe) o un inhibidor de la proproteína convertasa subtilisina/kexina tipo 9 (PCSK9) como el evolucumab y alirocumab ^6^^,^^29^^,^^32^.

Ningún estudio ha demostrado el beneficio del bloqueo del sistema renina-angiotensina en pacientes con SCC sin disfunción ventricular, por lo que este tratamiento no se considera indicado, salvo sea necesario para el control de la presión arterial ^5^^,^^6^^,^^27^^,^^33^.

### Nuevas terapias

Durante la última década han surgido nuevos grupos farmacológicos que han probado contribuir a reducir aun más el riesgo cardiovascular como el canakinumab, un inhibidor de la interleucina 1β, que toma en consideración el componente inflamatorio de la enfermedad ateroesclerótica y ha demostrado reducir la tasa de eventos cardiovasculares en pacientes con infarto de miocardio previo y PCR elevado ^34^.

El etilo de icosapento, un ácido graso omega 3 altamente purificado, que reduce el nivel de triglicéridos en sangre, demostró en el estudio REDUCE-IT disminuir la incidencia de eventos cardiovasculares en pacientes con enfermedad cardiovascular (incluyendo SCC) que presentaban hipertrigliceridemia a pesar de recibir terapia con estatinas, beneficio que antes no fue probado por el uso de otros reductores del nivel de triglicéridos como los fibratos o la niacina ^6^^,^^7^^,^^35^.

**Figura 1:**
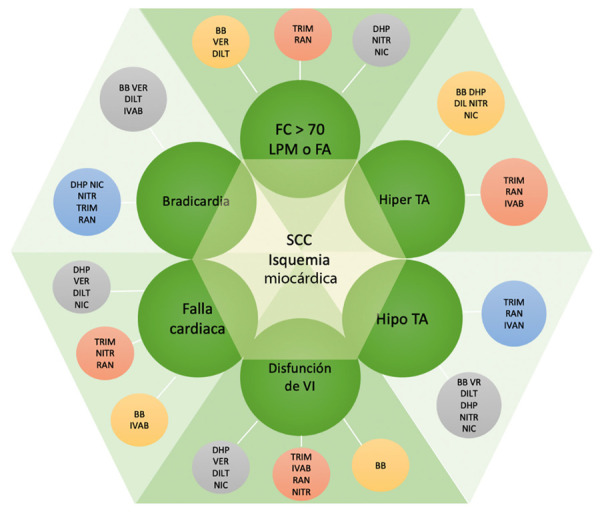
Diagrama de diamante en el manejo médico de la isquemia miocárdica y posibles combinaciones de acuerdo con las comorbilidades del paciente

Por último, en pacientes diabéticos con SCC, en quienes el riesgo de presentar un evento cardiovascular es alto, se ha estudiado el uso de los inhibidores del cotransportador 2 de sodio-glucosa (empaglifozina, canaglifozina, dapaglifozina) y análogos del péptido-1 humano, similar al glucagón (liraglutide) con los cuales se ha evidenciado una reducción significativa en la incidencia de infarto de miocardio, ACV y muerte cardiovascular al compararlo con un grupo placebo ^6^^,^^36^**(**Figura 1**).**

BB= beta bloqueadores. DHP= bloqueadores de calcio dihidropiridínicos. DILT= diltiazem. FC= frecuencia cardiaca. IVAB= Ivabradina. NIC= nicorandil. NITR= nitratos. RAN= ranolazina. SCC= síndrome coronario crónico. TRIM=trimetazidina. VER= verapamilo. Adaptado y traducido de Ferrari R, Camici PG, Crea F, et al. (29)

## Estudios clínicos sobre tratamiento médico óptimo conservador versus manejo invasivo inicial

**Figura 2:**
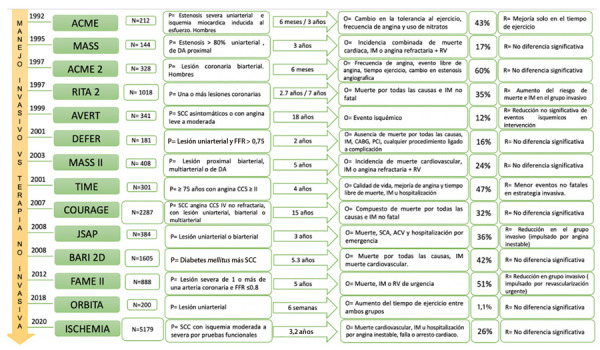
Principales ensayos clínicos en síndrome coronario crónico sobre beneficio del manejo médico óptimo de inicio versus manejo invasivo inicial

La indicación de terapia médica óptima (TMO) en SCC, también descrita como manejo conservador o no invasivo, ha demostrado tener un beneficio similar al uso del manejo invasivo. Diversos estudios no encontraron diferencia significativa en la incidencia de infarto de miocardio (IM), hospitalización, desarrollo de eventos adversos o incluso mortalidad ^7^^,^^24^^,^^25^**(**Figura 2**).**

La flecha horizontal en cada fila representa el seguimiento en años del estudio, el cuadro con el número absoluto en porcentaje (%) representa la cantidad de pacientes en el grupo de manejo médico óptimo inicial que fue a revascularización (quirúrgico y/o percutánea) durante el seguimiento del estudio. N= tamaño de muestral. P= población. O= variable principal de estudio. R= resultados.

Uno de los primeros estudios (MASS) sobre el impacto clínico del tratamiento no invasivo fue desarrollado en 1995. Este estudio comparó el uso de angioplastia con balón (APB) versus cirugía de revascularización (CRV) versus tratamiento médico (TM) en pacientes con angina estable y enfermedad coronaria severa (ECS) proximal de la arteria descendente anterior (DA) demostrando similar beneficio en la incidencia de muerte e IM ^37^^,^^38^. Paralelamente, el estudio ACME en pacientes con ECS uniarterial describe en el grupo invasivo un mayor alivio de angina a largo plazo, resultados que contrastaron con un estudio de similares características (ACME II) en pacientes con ECS biarterial donde no hubo diferencia significativa en el tiempo libre de angina, duración del ejercicio y mejoría de calidad de vida ^39^^-^^41^.

Con el surgimiento de los *stents* metálicos se elaboró el ensayo clínico RITA - 2 ^(42, 43)^ que adicionó al grupo de intervención invasiva por APB el uso de *stent* metálico; observando mejoría de síntomas, pero con un incremento de la mortalidad en el grupo posangioplastía. Asimismo, el estudio MAS II que comparó la angioplastía con *stent* metálicos vs. CRV vs. TMO en pacientes con enfermedad coronaria severa proximal biarterial, multiarterial o DA, evidenció una menor incidencia de muerte, IM y menor necesidad de revascularización en pacientes con TMO comparado a PCI. No obstante, el seguimiento a cinco años observó que el grupo CRV tuvo una reducción significativa de 44% de eventos comparado a TMO ^44^^,^^45^.

A finales del siglo pasado se agregó a la TM el uso de estatinas por mostrar un beneficio significativo en pacientes con SCC, así un estudio publicado en 1999 (AVERT) comparó el uso de manejo habitual más terapia agresiva con estatinas versus angioplastia con manejo habitual, observando una disminución importante en la incidencia de eventos isquémicos y aumento del tiempo libre de evento en el grupo de las estatinas. La era posestatinas marcó un hito en el manejo médico y mayor evidencia sobre el uso de TMO como terapia inicial en el SCC ^46^.

Por otro lado, el uso de reserva fraccional de flujo (RFF) demostró su utilidad como apoyo en la indicación de angioplastia, evidenciando en el estudio DEFER que la intervención percutánea a lesiones con un valor < 0,75, sin evidencia de isquemia previa, reducía el riesgo de eventos mayores; sin embargo, este brazo del estudio no fue comparado con el manejo conservador. Posteriormente, el ensayo clínico FAME II estudió 1220 pacientes y comparó el manejo percutáneo inicial versus el TMO solo, observando una diferencia significativa en beneficio del grupo invasivo sobre el compuesto de mortalidad, IM y revascularización de urgencia; sin embargo, al analizar independientemente la variable de muerte o IM en ambos grupos no se obtuvo diferencia significativa, por lo que los resultados del evento primario podrían estar impulsados por el aumento de revascularización de urgencia en el grupo no invasivo, evento que no demuestra implicancia clínica directa sobre la mortalidad o el IM ^47^^,^^48^.

El estudio COURAGE ^25^, uno de los primeros ensayos que incluyó la angioplastía con *stent* medicado en un pequeño porcentaje de la muestra, estudió 2287 pacientes con SCC y angina CCS IV no refractaria a TMO (estabilizados farmacológicamente) que poseían lesión coronaria severa (>80% o >70% con evidencia de isquemia en la misma área), todos los pacientes recibieron tratamiento antiagregante plaquetario, manejo hipolipemiante agresivo y manejo antiisquémico en combinación o no con IECA o ARA II como prevención secundaria. Los pacientes fueron estudiados y asignados aleatoriamente a los dos grupos de estudio manejo médico y manejo invasivo. En el seguimiento a cinco años del total de pacientes, 70% lograron disminuir significativamente el nivel de LDL; 64% presentaron buen control de presión sistólica, y 45% tuvieron un adecuado valor glicémico; se observó, además, una reducción significativa de angina, probablemente atribuible al uso de TMO indicado en ambos grupos. El evento primario compuesto de muerte e IM no fatal no fue significativamente diferente, ni tampoco hubo diferencia en los eventos secundarios como hospitalización por síndrome coronario agudo (SICA), ACV o muerte cardiaca. La revascularización en el grupo no invasivo fue realizada en el 32% de casos y estuvo indicada ante la presencia de angina refractaria a manejo optimo y/o empeoramiento de la isquemia.

A pesar de que la evidencia no encontró diferencias en la mortalidad, riesgo de IM y otras variables de relevancia clínica, en pacientes con manejo invasivo versus tratamiento médico conservador, el alivio de la angina es un factor importante en la mejoría de la calidad de vida del paciente coronario estable; dicha reducción sintomática ha sido descrita con mayor prevalencia en pacientes que tuvieron un manejo invasivo inicial ^24^^,^^25^. Bajo esta premisa, Al - Lamee *et al*. estudiaron sobre el posible efecto placebo del manejo invasivo en los estudios previos, y así fue como desarrolló el estudio ORBITA ^49^, un ensayo clínico aleatorizado doble ciego multicéntrico que incluyó 200 pacientes sintomáticos con ≥1 lesión coronaria severa revascularizable, los cuales recibieron TMO durante seis semanas y, posteriormente, fueron intervenidos para evaluación angiográfica anatómica y funcional bajo sedoanalgesia; luego de ello fueron distribuidos aleatoriamente en dos grupos: angioplastía con DES y placebo. Los resultados evidenciaron que no hubo diferencia significativa en ambos grupos sobre el incremento de tiempo de ejercicio, cambios en el segmento ST o consumo de oxígeno al ejercicio, ni sobre la mejoría clínica de angina; asimismo, ningún paciente falleció durante el estudio y solo 1,1% de los pacientes en el grupo conservador fueron revascularizados durante el seguimiento.

Al ser el primer ensayo clínico de su tipo (ciego) demostró que el manejo invasivo no incrementa el tiempo de ejercicio, ni mejora la angina más allá del efecto placebo, creando controversia sobre el manejo basado en isquemia y el impacto clínico de su resolución sobre la mortalidad, riesgo de IM y alivio sintomático. Aun así, los hallazgos de este y diversos estudios no sugieren que todo paciente con SCC no deba ser manejado por angioplastía sino, más bien, abogan por el uso adecuado del TMO inicial y la indicación prudente de angioplastía en pacientes con angina severa de inicio o refractaria a tratamiento médico. Hay que mencionar que siendo un estudio pionero debido a su característica de ensayo doble ciego, una limitante descrita es el número de pacientes estudiados muestra que en estudios próximos debe ser extendida en cantidad ^(^^7^^,^^49^.

Los pacientes con SCC estratificados como de alto riesgo (enfermedad coronaria severa multiarterial y/o lesión severa proximal de DA) tienen una elevada probabilidad de presentar SICA, angina inestable o algún evento fatal. Se tiene registro sobre el beneficio del manejo conservador inicial en esta población, como es el caso de los estudios MASS I y MASS II, donde se observó que tanto el TMO aislado como la angioplastia presentaban resultados similares en eventos mayores en pacientes con lesión coronaria severa multiarterial incluyendo DA proximal ^33^^,^^40^. En los últimos años esta premisa ha ganado peso debido al desarrollo de nuevas pruebas diagnósticas y la aparición de mejores estrategias terapéuticas en el SCC.

El estudio ISCHEMIA ^24^ es uno de los ensayos contemporáneos más grandes y mejor diseñados sobre la disyuntiva del manejo médico inicial versus invasivo en pacientes de alto riesgo. Este ensayo clínico aleatorizado incluyó a pacientes con SCC con evidencia de isquemia moderada a severa, definida como isquemia ≥ 10% de miocardio por prueba de perfusión nuclear, hipocinesia o acinesia ≥3/16 segmentos miocárdicos por ecocardiografía de estrés, isquemia ≥ 12% de miocardio y/o compromiso ≥3/16 segmentos miocárdicos por resonancia magnética o prueba de esfuerzo positiva severa. La distribución de la muestra fue aleatorizada (1:1) para manejo conservador no invasivo o manejo invasivo (angioplastia o CRV). Todos los pacientes tuvieron características basales similares. La angioplastia fue la intervención más utilizada en el grupo invasivo y el 21% de pacientes en el grupo conservador necesitaron angioplastia durante el seguimiento. A los cinco años de seguimiento no se obtuvo diferencia significativa entre los grupos sobre el estimado acumulado de eventos mayores (muerte cardiovascular, IM u hospitalización). Por otro lado, el análisis de variables secundarias evidenció una mayor incidencia de infartos periprocedimiento en el grupo invasivo y una mayor incidencia de infarto espontáneo en el grupo conservador; aun así, la diferencia en la presentación del tipo de infarto en cada grupo no tuvo un impacto en la mortalidad al final del estudio; se observó, además, un incremento en la hospitalización por falla cardiaca en el grupo invasivo con menor incidencia de hospitalización por angina inestable, probablemente asociado a lesión renal aguda e infarto relacionado a procedimiento. El análisis por subgrupos demostró que no existe diferencia significativa de muerte cardiovascular, IM u hospitalización ante la presencia de lesiones multiarteriales o lesión proximal de DA.

Si bien es cierto, existe evidencia que puede poner en controversia estos hallazgos, como la descrita por Miller *et al.*^50^ en un estudio observacional de gran tamaño, donde evidenciaron que los pacientes con SCC e isquemia > 15 % se beneficiaban de una estrategia de revascularización temprana; y por otro lado, Azadari *et al.* ^(^^21^, observaron mejoría en la sobrevida de pacientes con SCC sometidos a revascularización temprana e isquemia > 10,2%; la presencia de limitaciones metodológicas en ambos estudios disminuye su validez interna (falta de aleatorización de los pacientes, la ausencia de control del uso de tratamiento médico óptimo, la presencia de diferencias significativas entre las características basales de los grupos de estudio y la inclusión en la muestra de pacientes con enfermedad severa de TCI y angina refractaria a tratamiento, las cuales son indicaciones directas de revascularización temprana recomendadas en las guías actuales de manejo y son criterios de exclusión en la población de estudio de los ensayos citados anteriormente).

El estudio BARI 2D ^52^ con seguimiento a cinco años comparó a pacientes diabéticos con SCC y una o más lesiones coronarias severas que recibieron TMO conservador versus manejo invasivo inicial; demostrando tener la misma incidencia en mortalidad, IM o ACV; asimismo, no hubo cambios en los resultados al analizar el uso de tratamiento sensibilizador de insulina o proveedor de insulina en ambos grupos. 

Un estudio paralelo al ISCHEMIA (ISCHEMIA CKD) ^53^ realizó el mismo análisis comparativo en pacientes con enfermedad renal crónica e isquemia moderada a severa, sin evidenciar diferencias en el evento mayor; por el contrario, se reportó una mayor tasa de complicaciones periprocedimiento, ACV y diálisis en el grupo invasivo. Por último, el ensayo clínico TIME que evaluó a pacientes >75 años con angina CCS II-IV, no reportó diferencias en la calidad de vida, muerte o IM entre ambos grupos ^54^.

**Figura 3:**
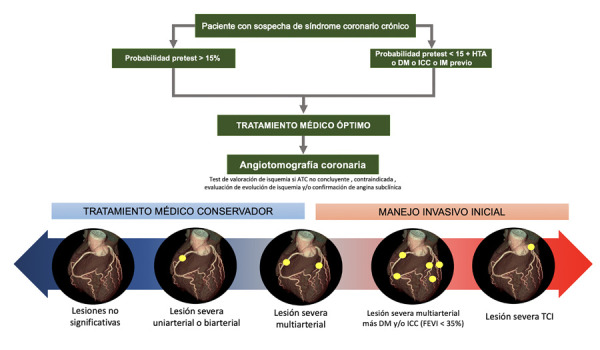
Ilustración central del manejo del síndrome coronario crónico de acuerdo con la presencia de enfermedad coronaria ateroesclerótica, obstrucción intraluminal y comorbilidades

Por lo expuesto, la RVM inicial de rutina no se encuentra asociada a mejoría en la supervivencia, presencia de IM, falla cardiaca o ACV, al compararla con el manejo médico conservador inicial en pacientes con SCC; información que es compartida por Bangalore *et al*. en un metaanálisis recientemente publicado donde se analiza la evidencia acumulada de varios estudios y sugieren un abordaje multidisciplinario que considere el riesgo versus el beneficio del abordaje invasivo en estos pacientes. Entre los factores que limitan el TMO conservador se encuentran la angina refractaria a TMO, angina CCS IV de inicio, lesión severa de TCI, enfermedad multiarterial asociada a diabetes *mellitus*, empeoramiento de la isquemia a corto plazo, disfunción ventricular significativa, entre otros ^55^**(**Figura 3**).**

ATC = angiotomografía computarizada. DM= diabetes mellitus. ICC = insuficiencia cardiaca congestiva. IM = infarto de miocardio. FEVI= fracción de eyección del ventrículo izquierdo. TCI = tronco común coronario izquierdo.

## Conclusiones

El manejo médico conservador comparado al manejo invasivo inicial no han demostrado tener diferencias significativas en cuanto a la mortalidad por todas las causas e incidencia de IM, variables clínicas que constituyen el objetivo principal del manejo del SCC.

El TMO en SCC como prevención secundaria debe tener como objetivos un LDL<55, una presión arterial <130/80 mmHg, uso de antitrombóticos y el control de glicemia.

Los pacientes con SCC de alto riesgo deben de ser individualizados en su manejo ya que, si bien es cierto, la evidencia actual ha demostrado semejanza en la reducción de eventos cardiovasculares mayores en ambas formas de manejo, la decisión sobre la mejor estrategia terapéutica debe ser tomada por un equipo multidisciplinario *(Heart Team)* que considere otras variables relevantes como angina refractaria, progresión significativa de la isquemia, viabilidad de manejo (anatómico) de la lesión y comorbilidades (diabetes *mellitus*, enfermedad renal crónica, edad, historia clínica previa, disfunción ventricular significativa, etc.).

Se debe brindar información al paciente con SCC sobre las ventajas de un manejo conservador inicial, incluso en aquellos con alto riesgo, así como los beneficios del cumplimiento de la TMO y la necesidad de un seguimiento clínico estricto evaluando conjuntamente la sintomatología y la calidad de vida. 
