# Supplementary material for: Should I treat a high-risk chronic coronary syndrome in an invasive way from the beginning? No, in most cases
Source: Arch Peru Cardiol Cir Cardiovasc. 2020 Dec 31;1(4):229–39. [Article in Spanish] doi: 10.47487/apcyccv.v1i4.98 (PMC10804823; doi:10.47487/apcyccv.v1i4.98)
Supplement: Related texts

# Reply: Respuesta a Carrasco *et al*

**Authors:** César Ortiz Zegarra, Piero Custodio Sánchez, Paol Rojas de la Cuba, Ricardo Coloma Araniya, Christian Nolte Rickards

Este artículo presenta estudios de alta calidad metodológica, pero con validez externa cuestionable en nuestro medio, pues son realizados en poblaciones con sistemas de salud donde la atención en casos de eventos isquémicos agudos es mucho más accesible que en nuestro medio. 

Se ha mencionado estudios con manejo invasivo utilizando dispositivos que actualmente están obsoletos como stent metálicos o angioplastias solo con balón y por consiguiente no pueden ser aplicados a nuestra realidad actual, pues en Perú solo se realiza intervencionismo coronario con stent medicados, inclusive en la mayoría de casos utilizando los de última generación como lo recomiendan las actuales guías internacionales de revascularización.

Se menciona el estudio COURAGE donde casi la tercera parte (32%) de los que recibieron tratamiento médico fueron sometidos a angioplastia coronaria durante el seguimiento y la mayoría durante el primer año. Asimismo, la mortalidad incluyó todas las causas, lo cual podría considerarse un sesgo para ver la influencia del tratamiento invasivo en el pronóstico.

El estudio ORBITA solo incluye a pacientes con enfermedad coronaria uniarterial severa determinada por angiografía, y el tiempo de seguimiento fue corto: solo 6 semanas. Otro artículo que se menciona es el ISCHEMIA, pero no menciona el subestudio que incluyó pacientes de alto riesgo con fracción de eyección moderadamente disminuida o con historia de falla cardiaca, que si se beneficiaron con un manejo inicial invasivo al presentar menor eventos cardiovasculares mayores, hospitalizaciones por angina inestable o falla cardiaca. Además, es importante recalcar que en el estudio ISCHEMIA el tamaño de muestra se redujo en más de 25%, el periodo de seguimiento disminuyó de 4 a 3,2 años, se incluyeron a pacientes de bajo riesgo en las pruebas de isquemia (12%) y la reducción de angina fue de 50% en el grupo de tratamiento invasivo frente a 20% en el grupo de manejo conservador al año de seguimiento. Todos estos aspectos hacen inadecuado analizar la implicancia de este estudio en nuestra realidad. 

Otro aspecto para considerar es que además del desarrollo de las opciones terapéuticas para manejo conservador de síndrome coronario crónico, también ha habido una gran revolución en nuevos dispositivos utilizados en el manejo invasivo con stent liberadores de fármacos de última generación, imágenes intracoronarias, pruebas de isquemia invasivas y diversos instrumentos que permiten disminuir riesgos y aumentar la tasa de éxito en revascularización coronaria.
